# Gaussian process latent variable models-ANN based method for automatic features selection and dimensionality reduction for control of EMG-driven systems

**DOI:** 10.3389/frai.2025.1506042

**Published:** 2025-01-22

**Authors:** Maham Nayab, Asim Waris, Muhammad Jawad Khan, Dokhyl AlQahtani, Ahmed Imran, Syed Omer Gilani, Umer Hameed Shah

**Affiliations:** ^1^National University of Science and Technology, Islamabad, Pakistan; ^2^Department of Electrical Engineering, School of Engineering, Prince Sattam Bin Abdul Aziz University, Al-Kharj, Saudi Arabia; ^3^Department of Biomedical Engineering and Artificial Intelligence Research Center, College of Engineering and Information Technology, Ajman University, Ajman, United Arab Emirates; ^4^Abu Dhabi University, Abu Dhabi, United Arab Emirates; ^5^Department of Mechanical Engineering and Artificial Intelligence Research Center, College of Engineering and Information Technology, Ajman University, Ajman, United Arab Emirates

**Keywords:** ANN, dimensionality reduction, feature selection, GPLVM, myoelectric control, PCA

## Abstract

Electromyography (EMG) signals have gained significant attention due to their potential applications in prosthetics, rehabilitation, and human-computer interfaces. However, the dimensionality of EMG signal features poses challenges in achieving accurate classification and reducing computational complexity. To overcome such issues, this paper proposes a novel approach that integrates feature reduction techniques with an artificial neural network (ANN) classifier to enhance the accuracy of high-dimensional EMG classification. This approach aims to improve the classification accuracy of EMG signals while substantially reducing computational costs, offering valuable implications for all EMG-related processes on such data. The proposed methodology involves extracting time and frequency domain features from twelve channels of EMG signals, followed by dimensionality reduction using techniques such as PCA, LDA, PPCA, Lasso and GPLVM, and classification using an ANN. Our investigation revealed that LDA is not appropriate for this dataset. The dimensionality reduction models did not have any significant effect on the accuracy, but the computational cost decreased significantly. In individual comparisons, GPLVM had the shortest computational time (29 s), which was significantly less than that of all the other models (*p* < 0.05), with PCA following at approximately 35 s and Relief at approximately 57 s, while PPCA took approximately 69 s, and Lasso exhibited higher computational costs than all the models but lower computational costs than did the original set. Using the best-performing features, all possible sets of 2, 3, 4 and 5 features were tested, and the 5-feature set exhibited the best performance. This research demonstrates the effectiveness of dimensionality reduction and feature selection in improving the accuracy of movement recognition in myoelectric control.

## 1 Introduction

Electromyography (EMG) is a powerful tool that has revolutionized research and healthcare in the field of applied health. It is a diagnostic technique that is used to record and evaluate the electrical activity of skeletal muscle. The importance of EMG signals in research and healthcare cannot be underestimated. They play a vital role in understanding the neuromuscular system and can help diagnose and sometimes even predict various neuromuscular disorders, such as Parkinson’s disease (Adem et al., 2022), muscular dystrophy (Quijano-Roy et al., 2004), myasthenia gravis (Baruca et al., 2016), and amyotrophic lateral sclerosis (Sonoo et al., 2009). These signals help clinicians identify the specific muscles affected by the disorder and assess the severity of the conditions (Dai et al., 2022; Nazmi et al., 2016). EMG signals are also used to guide treatments such as physical therapy, electrical stimulation, and surgery. In rehabilitation, EMG signals can be used to evaluate the effectiveness of physical therapy interventions and monitor progress (Dai et al., 2022). EMG signals can provide real-time feedback on muscle activation patterns and help patients learn to activate specific muscles or muscle groups correctly. This feedback can improve motor control and enhance the effectiveness of rehabilitation programs, leading to better outcomes for patients (Nazmi et al., 2016). In myoelectric control, EMG signals are used to control prosthetic devices. These signals are recorded from residual muscles in the amputated limb and transferred to the prosthetic device, allowing the user to control the device’s movements. The EMG signals can be processed in real time to translate the user’s muscle activity into prosthetic movement, providing a more natural control interface. This can significantly improve the functionality and usability of prosthetic devices, allowing users to perform daily activities more effectively (Chen et al., 2023).

In the field of EMG signal processing, feature extraction is the most crucial step for extracting useful and important information from complex signals. Numerous studies have been conducted to identify new features that can belong to the time domain, frequency domain, or time-frequency domain (Chen et al., 2023; Abbaspour et al., 2020; Phinyomark et al., 2014; Chen et al., 2006; Too et al., 2019b; Danneels et al., 2002; Too et al., 2019c; Naik et al., 2016; Sachin, 2015; Rehman, 2018; Waris et al., 2018; Jochumsen et al., 2018; Zia ur Rehman et al., 2018; Waris et al., 2019; Daffertshofer et al., 2004). However, most of the features are redundant, and using redundant features together could decrease the classification accuracy. Therefore, it is crucial to select appropriate features to achieve optimal performance. Researchers have made significant progress in identifying suitable feature sets and studying their effects on classification accuracy in recent years. The importance of feature reduction lies in its ability to address the issue of high dimensionality, which can lead to overfitting, reduced classification accuracy, and increased computational complexity. Feature reduction techniques can help overcome this problem by reducing the dimensionality of the feature space, thereby improving classification accuracy and reducing computational complexity (Too et al., 2019a; Phinyomark et al., 2012; Phinyomark et al., 2012). Several dimensionality reduction techniques can effectively reduce the number of features while retaining the most informative features. In Zhang et al. (2012), implemented PCA on their EMG data consisting of 512 samples and observed a success rate of 99.8%, indicating the effectiveness of PCA in accurately controlling multiple finger movements using EMG signals. In Yang et al. (2013), employed the wavelet packet transform to extract features from sEMG signals and subsequently applied nonparametric discriminant analysis (NDA) for feature reduction together with a support vector machine (SVM) for classification. Their approach achieved an impressive average accuracy of 98 to 99% per subject. These results demonstrate the potential of using a combination of these methods for accurate EMG pattern recognition. In Junior et al. (2020), compared various techniques of feature reduction for surface electromyography (sEMG) signals obtained from an armband. They employed both feature selection and dimensionality reduction methods, along with seven classifiers, to recognize six different gestures performed by 13 subjects. The researchers found that the highest accuracy of 94% was achieved by combining the support vector machine classifier with dimensionality reduction. In Chu et al. (2007), compared LDA, PCA, NLDA, and self-organizing feature map (SOFM) methods for the classification of features extracted from four-channel EMG signals using WPT. They found that LDA had the best accuracy and real-time performance but required calculating the within-class scatter matrix, which may be singular if there is high feature redundancy. To address this issue, a combination of PCA and SOFM was proposed, which showed improved performance over PCA alone in terms of myoelectric control. This highlights the importance of using appropriate feature reduction methods for improving the accuracy of sEMG signal classification.

**Figure 1:**
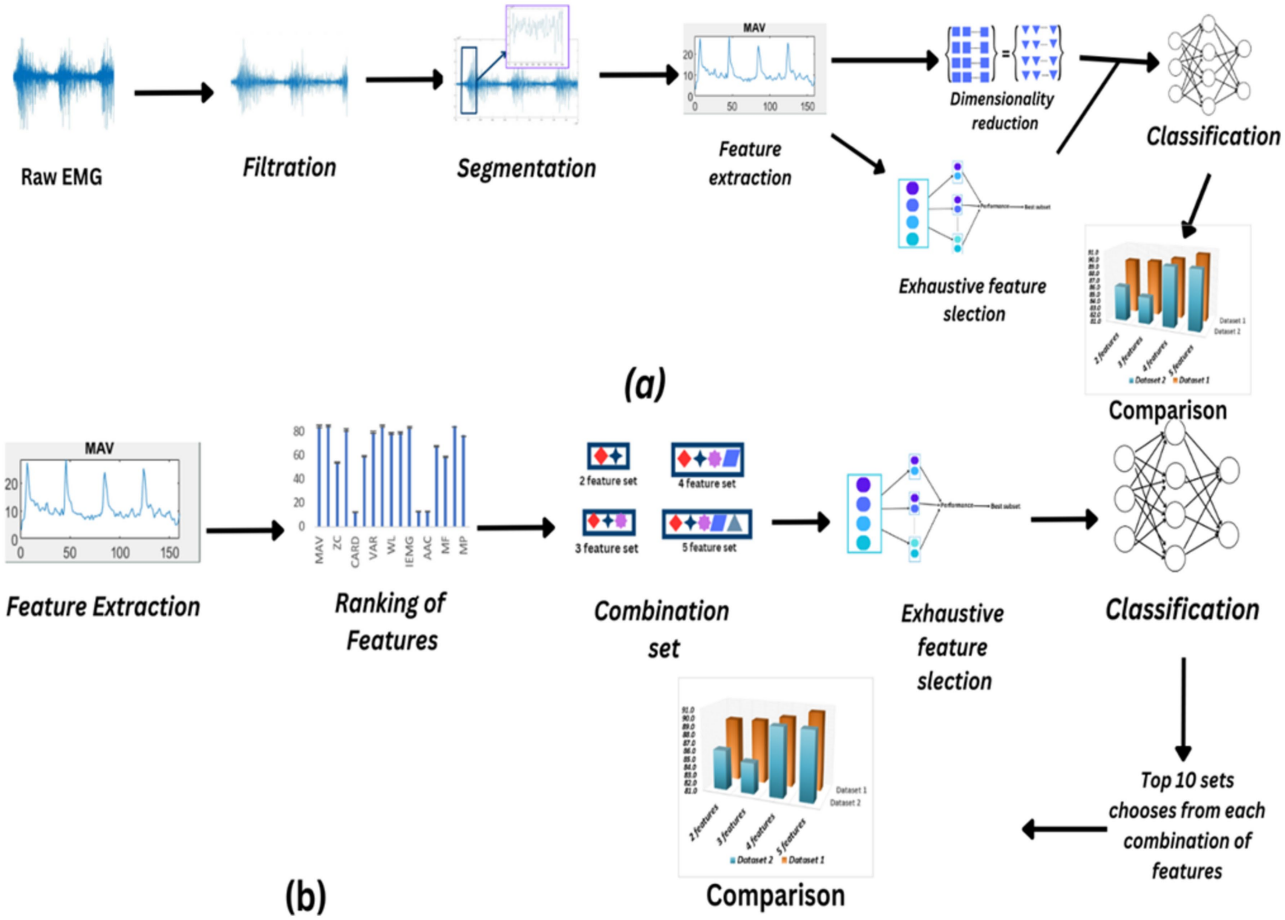
**(A)** Flowchart illustrating the process of filtering, segmenting, dimensionality reduction, and exhaustive feature selection techniques applied to raw EMG data, with subsequent comparison of the resulting sets. **(B)** Feature extraction, ranking, and selection process leading to the creation of different feature combinations for classification, followed by comparison using ANOVA to determine optimal feature sets.

Most previous studies have focused on subject-specific EMG classification, whereas this study emphasizes generalized movement classification, which is particularly relevant in robotics and prosthetics. In this study, self-acquired data from an online database is used. The aim of this study is to enhance the performance of classifiers for myoelectric control in generalized devices, including prosthetic limbs, exoskeletons, rehabilitation robots, and assistive robotic systems, while identifying optimal feature sets. Six dimensionality reduction techniques such as Principal Component Analysis (PCA), Linear Discriminant Analysis (LDA), Probabilistic PCA (PPCA), Gaussian Process Latent Variable Models, Lasso and Relief were applied and their effect on classification accuracies and computational cost is observed. While optimal feature sets are identified using exhaustive feature selection. The most suitable feature sets are identified from 40 well-known electromyography features. The comparison between feature selection methods and dimensionality reduction techniques focused on model accuracy and computational time. Overall, the work emphasizes generalized movement classification rather than subject-specific approaches. Figure 1 illustrates the study’s workflow.

## 2 Materials and methods

### 2.1 Data acquisition

This research utilizes two different datasets. Dataset 1 utilizes the online Ninapro database, specifically the second database known as DB2 (Atzori et al., 2014). The dataset consists of EMG signal data from 40 healthy individuals recorded using the Delsys Trigno Wireless EMG system with a sampling rate of 2 kHz. This work is based on the data from 10 of these subjects and examines EMG signals during six distinct movements: abduction and flexion of all fingers (AF, FF), wrist extension (WE), wrist radial deviation (WRD), wrist ulnar deviation (WUD), and wrist extension with a closed hand (WE). Each movement was repeated six times, with each repetition lasting for 5 s and 3 s of rest between repetitions.

The second dataset was EMG signals collected from ten able-bodied subjects aged between 20 and 35 years (three men/seven women,). The procedures were in accordance with the Declaration of Helsinki and the local ethics committee of the National University of Sciences and Technology, Islamabad, Pakistan (approval number ref. # BMES/REC/22/027). Subjects provided their written, informed consent prior to the experimental procedures. The subjects had no history of upper extremity or other musculoskeletal disorders. Ot Bioelectronica was used for recording EMG signals at a 2 kHz sampling rate. Eight pairs of differential electrodes were positioned below the radio humeral joint at equal intervals; two pairs were positioned on the flexor and extensor digitorium and one pair was placed on the biceps brachii. Six movements were required of the subjects:

- wrist extension (WE)
- wrist radial deviation (WRD)
- wrist ulnar deviation (WUD)
- wrist extension with a closed hand (WE)
- Abduction of all fingrers (AF)
- Fingers Flexed together in a wrist (FF)

**Figure 2:**
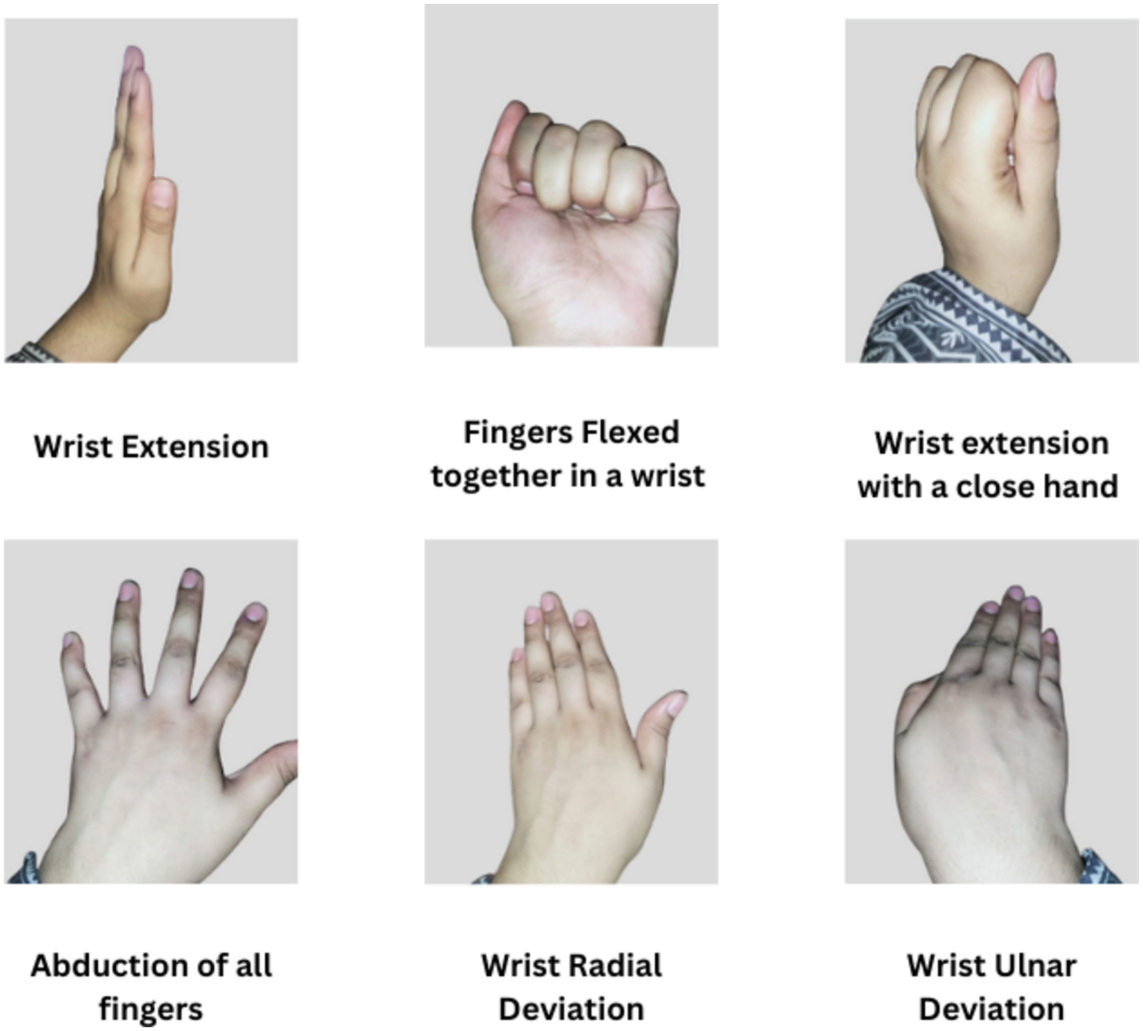
Movements performed by subjects.

Figure 2 illustates the movements performed by subjects. Every movement was performed four times, with a three-second break in between each five-second repetition.

### 2.2 Pre-processing and feature extraction

Initially, the raw EMG data were preprocessed by applying denoising filters. A notch filter was applied to remove 50 Hz powerline interference, and a fourth-order Butterworth bandpass filter was utilized to permit frequencies within the range of 20–500 Hz (Naik et al., 2016). After filtration, the data were segmented into overlapping segments of 250 ms with 50% overlap. There are three different types of features of EMG signals: the time domain, frequency domain, and time-frequency domain. In this work, thirty-four time domain and six frequency domain features were extracted (Abbaspour et al., 2020; Phinyomark et al., 2014; Chen et al., 2006; Too et al., 2019b; Danneels et al., 2002; Too et al., 2019c; Naik et al., 2016; Sachin, 2015; Rehman, 2018; Waris et al., 2018; Jochumsen et al., 2018; Zia ur Rehman et al., 2018; Waris et al., 2019; Spiewak, 2018). The features are listed in Table 1.

**Table 1:** Features.

Sr No	Features	Sr No	Features
1	Integrated Emg (iEMG)	21	Maximum Fractal Length (MFL)
2	Root Mean Square (RMS)	22	Log Difference Absolute Mean Value (LDAMV)
3	Variance (VAR)	23	Log of coefficient of variation (LCOV)
4	Waveform Length (WL)	24	Wilson Amplitude (WA)
5	Zero Crossing (ZC)	25	Average Amplitude Change (AAC)
6	Slope Sign Change (SSC)	26	Coefficient Of Variation (CV)
7	Mean Absolute Deviation (MAD)	27	Hjorth Mobility (Hmob)
8	Simple Square Integral (SSI)	28	Absolute Value Of The Summation Of Square Root (ASS)
9	Average Energy (AE)	29	Approximate Entropy (AE)
10	Skewness (Skew)	30	Cardinality (CARD)
11	Modified Mean Absolute Value 1 (MMAV1)	31	Interquartile Range (IQ)
12	Modified Mean Absolute Value 2 (MMAV2)	32	Integrated Absolute Value (IAV)
13	3RD Temporal Moment (TM3)	33	Kurtosis (Kurt)
14	4TH Temporal Moment (TM4)	34	Maximum Energy (ME)
15	5TH Temporal Moment (TM5)	35	Peak Frequency (PF)
16	Standard Deviation (std)	36	Mean Power (MP)
17	V Order (Vo)	37	Mean Frequency (MF)
18	Log Detector (LD)	38	Frequency Ratio (FR)
19	Difference Absolute Mean Value (DAMV)	39	Total Power (TP)
20	Difference Absolute Standard Deviation Value (DAVSR)	40	Modified Mean Frequency (Mmf)

### 2.3 Feature ranking

After feature extraction, each feature was ranked individually based on its performance. For this, one feature at a time was used as input to the neural network classifier, and its classification accuracy was evaluated. Features that achieved an accuracy above the 80% threshold were considered the best performing. This ranking process was performed using data from a single subject to assess the individual feature performance. All the other features with accuracies less than the threshold were eliminated from data set.

### 2.4 Dimensionality reduction techniques

After feature ranking, all features that performed above the threshold were extracted from all subjects across all movements. A generalized approach was applied, where data from all subjects were combined and used to classify the movements, rather than performing classification for each subject individually. The dataset, consisting of 10 subjects, 5 movements, 12 muscles, and 15 features per muscle signal, resulted in a high-dimensional and complex data structure. This led to decreased classification accuracy and increased classifier processing time. Several existing works (Too et al., 2019a; Phinyomark et al., 2012; Phinyomark et al., 2012) have reported the existence of many redundant features in EMG signals, which degrade signal classification accuracy. To increase the classification accuracy, dimensionality reduction techniques can be used. In this work, five dimensionality reduction techniques were employed, namely, principal component analysis, linear discriminant analysis, probabilistic PCA, least absolute shrinkage and selection, the Relief algorithm and Gaussian process latent variable models (GPLVMs).

PCA is a well-known unsupervised linear dimensionality reduction technique that enables the transformation of high-dimensional data into a lower-dimensional space while maintaining crucial information (Daffertshofer et al., 2004). The main goal of PCA is to identify the most important features that have high covariance and use them to transform data into a new vector space with uncorrelated features. The set of new features captures the main trends in key data from the original data, thus making it simpler to visualize and analyze it (Daffertshofer et al., 2004). Linear Discriminant Analysis (LDA) is a widely known supervised statistical technique used for classification and dimensionality reduction. It works by projecting high-dimensional data onto a linearly low-dimensional space that represents discriminative information in the data. This is done by trying to maximize class separability while minimizing within-class scatter. LDA finds applications in many areas such as image processing, speech recognition, bioinformatics etc. due to its simplicity and ease of computation which makes it widespread for various tasks in an analysis of data (Tharwat et al., 2017).

Probabilistic PCA on the other hand is an extension of principal component analysis and is also a linear dimensionality reduction technique. This method uses a probabilistic model to transform data through linear transformations from high dimensions to lower ones unlike the PCA. It assumes that there exists low dimensional subspaces subject to additional Gaussian noises that generate such observations. PPCA estimates this low-dimensional subspace as well as the linear transformation by adding Gaussian noise to a linear transformation of the low-dimensional subspace through expectation maximization algorithm (EM). The noise covariance matrix and model parameters are estimated alternately via the EM technique. The resulting low-dimensional representation of the data preserves its essential features (Tipping and Bishop, 1999).

The GPLVM is a nonlinear technique used for mapping high-dimensional data into a low-dimensional latent space. It works similarly to PPCA except that GPLVM is nonlinear. The GPLVM uses a nonlinear function, which is modeled as a Gaussian process (Lalchand et al., 2022). The mapping parameters between the latent and observed variables are treated as random variables with a prior distribution, allowing GPLVMs to capture complex relationships between the variables. The prior distribution is often chosen as a Gaussian distribution, which enables efficient computation via Gaussian process regression. By incorporating a prior distribution on the mapping parameters, GPLVMs can perform Bayesian inference to estimate the posterior distribution of the mapping parameters given the observed data, allowing for probabilistic estimates of the mapping parameters and uncertainty in the model.

Kira and Rendell (1992) introduced the Relief algorithm. It functions as an independent assessment method for feature selection. Relief computes a surrogate statistic for each feature, aiding in the estimation of feature ‘quality’ or ‘relevance’ to the target concept, such as predicting the endpoint value. These statistics are denoted as feature weights (weights of feature ‘A’) or, more informally, as feature ‘scores’, which span a continuum from poor to excellent performance (Kira and Rendell, 1992).

Least absolute shrinkage and selection operator (LASSO) is a popular technique used in machine learning and statistics for selecting a subset of features from a larger set of available features (Muthukrishnan and Rohini, 2016). The functions through adding a penalty toward the standard objective function of linear regression, which penalizes regression coefficients’ absolute magnitudes. What this does is to promote sparsity of the solution which means that many of the coefficients are driven to zero and in effect, only a subset of original features are selected. Lasso feature selection is particularly useful in high-dimensional datasets where there are more features than samples, as it can help identify most important predictors while discarding irrelevant or redundant ones. It can also be used for prevention of overfitting and improvement in interpretation by LASSO feature selection (Fonti and Belitser, 2017).

### 2.5 Exhaustive feature selection

Exhaustive feature selection is a brute-force approach used frequently in machine learning for systematically evaluating all possible feature combinations from a given dataset (Bouzoubaa et al., 2020). Unlike other methods for selecting features that rely on heuristic algorithms or statistical measures, exhaustive feature selection looks exhaustively through all possible feature subsets to identify one that optimizes a predefined criterion like model performance or predictive accuracy. Although exhaustive feature selection guarantees finding the optimal subset of features it becomes computationally expensive as the number of features increases making it impractical for datasets with a large number of features. Despite its computational cost, exhaustive feature selection is valuable in scenarios where model interpretability and the identification of the best-performing feature subset are paramount.

### 2.6 Classification

A feature set is constructed for movement classification following feature extraction. An ANN was used as a classifier in this evaluation. ANNs are a widespread machine learning method that can figure out intricate linkages and patterns. The composition and operation of the human brain serve as an inspiration for them. An ANN is made up of layers of connected nodes, or neurons. From the input layer, where data are introduced through one or more hidden layers, to the output layer, where predictions or classifications are formed, information travels through the network. Activation functions are used by each neuron to change the input data, and during training, weights—a measure of the strength of connections between neurons—are modified to reduce error between the expected and actual outputs. In this study, a three-layer ANN was used, with each layer containing 20 neurons. The activation function applied was the Rectified Linear Unit (ReLU), and the model was trained for 1,000 iterations. The same ANN was applied after all feature selection and dimensionality reduction methods were performed. All dimensionality reduction and feature selection methods were evaluated using the ANN. Classification accuracies were recorded both before and after dimensionality reduction and then compared to evaluate their performance.

In this study, we employed 5-fold cross-validation to evaluate the model’s performance. The dataset was divided into 5 equal folds, and for each iteration, one fold was used for testing while the remaining four folds were used for training. This process was repeated 5 times, ensuring that each fold served as the testing set once. The average performance across all folds was then reported, providing a more robust estimate of the model’s generalization capability.

### 2.7 Statistical analysis

For the dimensionality reduction techniques, the computational cost and accuracies were recorded. The pre-and post-reduction accuracies were compared to observe any significant differences. Similarly, all dimensionality reduction techniques were compared to evaluate which performed better in terms of both accuracy and computational cost. After exhaustive feature selection, the top 10 sets of all the combinations were compared by using statistical analysis. ANOVA was used to analyze and choose the best combination. The best feature sets were choosen based on the classification accuracies. One-way ANOVA was used to analyze the results and determine any statistically significant differences. Since the data followed a nominal distribution, ANOVA was an appropriate choice.

## 3 Results

**Figure 3:**
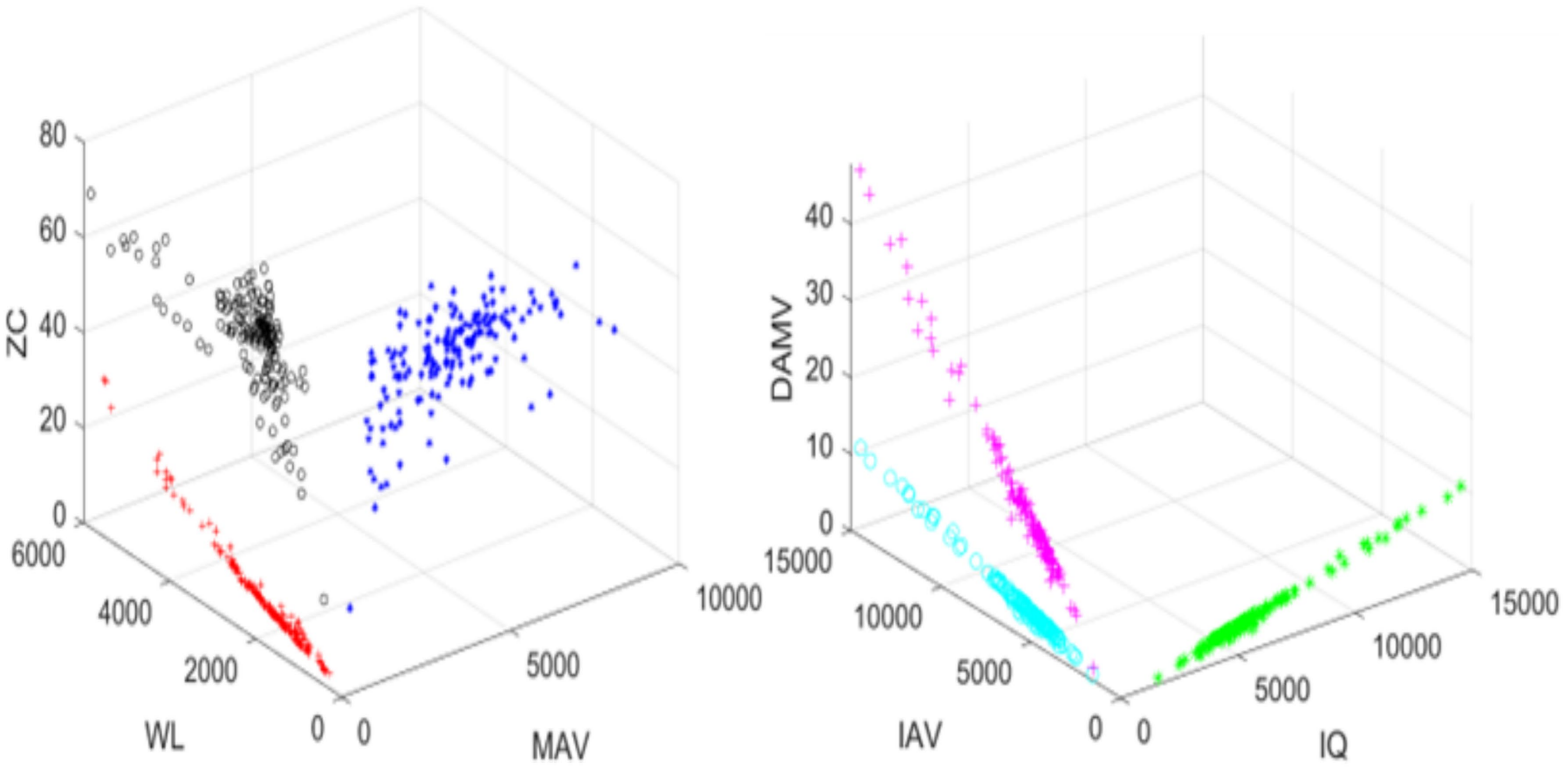
Scatter plot of EMG features for 1 movement.

To observe the similarities and importance of the extracted features, 3D scatter plots were generated. The scatter plots of DAMV, IAV, IQ, ZC, WL and MAV are shown in Figure 3. The scatter plots show that the majority of the data points are closely cluttered together, which indicates the presence of redundancy in the EMG data (Figure 3).

### 3.1 Feature ranking and elimination

**Figure 4:**
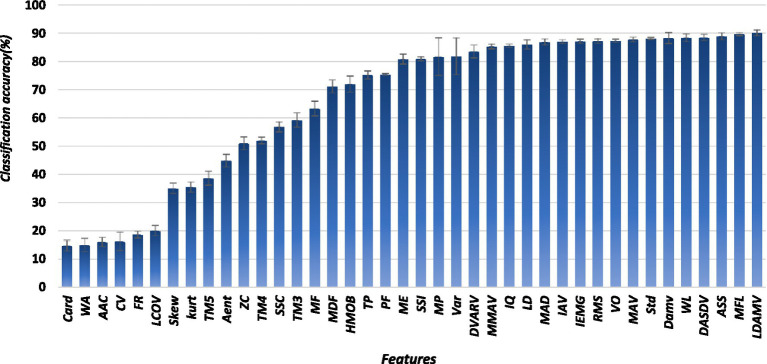
Feature wise classification accuracy.

All forty-one features were evaluated by using ANN. They were ranked based on their classification accuracies. LDAMV had the highest classification accuracy (89%), while cardinality and the WAM were the worst-performing features, with classification accuracies lower than 20%. Figure 4 shows classification accuracy of each individual feature.

Out of 40 features, all the features for which the classification accuracy was less than 85% were eliminated, and only 16 features remained. The remaining features were the modified mean absolute value, interquartile, mean absolute deviation, integral absolute value, integrated EMG, V order, mean absolute value, standard deviation, difference absolute mean value, waveform length, difference absolute standard deviation value, absolute value of the summation of square root, maximum fractal length, and log difference absolute mean value. The trend of feature performance was the same in both datasets.

### 3.2 Comparison of feature sets with different dimensionality reduction techniques

After conducting feature elimination, a feature vector comprising 16 features from 11 different channels and representing 6 distinct classes extracted from the EMG signals across 10 subjects was generated. Subsequently, various feature selection and reduction techniques were applied to this dataset, and their performance was evaluated based on computational cost and classification accuracy. Given the aim of developing a generalized model, data from all subjects were amalgamated.

Initially, an artificial neural network (ANN) was employed to assess the feature vector post elimination, yielding an accuracy of 88.7%. However, the computational time required for training and evaluating the ANN was approximately 115 s. LDA failed to perform on a large dataset such as this one. Subsequently, PCA was applied, resulting in an accuracy of 88.23% and a substantial reduction in computational time to 35 s (*p* < 0.05). Similarly, PPCA applied to the original feature vector improved the accuracy to approximately 90%, while reducing the computation time significantly to 69 s (*p* < 0.05).

When GPLVM was applied to the original feature vector, the accuracy remained relatively constant, yet the computation time decreased significantly to only 29 s (*p* < 0.05). Relief algorithm application resulted in an accuracy of 89.3% with a computation time reduction to 57 s (*p* < 0.05). Additionally, the Lasso application yielded an accuracy of 89%, accompanied by a notable reduction in computation time to 90 s (*p* < 0.05). One-way ANOVA showed no significant difference among the accuracies of all the tested techniques.

The feature selection methodologies had a negligible impact on the classification accuracy but had a considerable influence on the computational expense. Across the spectrum of models, a marked reduction in computational costs was evident compared to the original feature set. Notably, Lasso exhibited significantly diminished costs relative to the original dataset, albeit with higher expenses than other methodologies. PPCA demonstrated a reduction in computational overhead compared to both Lasso and the original set, albeit remaining higher than alternative approaches. Relief exhibited substantially reduced costs in comparison to Lasso, PPCA, and GPLVM, although it surpassed PCA in computational expenses. PCA demonstrated diminished computational costs across models, except for GPLVM, which emerged as the frontrunner with significantly reduced expenses compared to all counterparts. Figure 4 illustrates the classification accuracies and computational costs for each dimensionality reduction technique.

### 3.3 Exhaustive feature selection

Exhaustive feature selection was systematically applied to the datasets, employing sets comprising two, three, four, and five features. Through exhaustive exploration, all possible combinations of these feature sets were obtained, facilitating a comprehensive evaluation of performance based on classification accuracies. The computational expenditure associated with exhaustive feature selection surpassed that of all other models; however, this was justified by the significantly higher accuracies achieved. Following feature selection, the cost of model training was reduced to a mere second.

Furthermore, the best-performing sets comprising two, three, four, and five features are delineated in the subsequent section.

#### 3.3.1 Two-feature combinations

There are a total of 120 combinations of the 16 features, each of which contains 2 features. All feature sets were evaluated using an ANN. The classification accuracy ranged from 85.39 to 91.87%. The best-performing feature sets were chosen based on the classification accuracies. The top 10 sets of both datasets are depicted in Table 2. Their classification accuracies are plotted in Figures 6, 7.

**Table 2:** Optimal two-feature sets.

Sets	Features	
	Dataset 2	Dataset 2
Set 1	IAV, LDAMV	MAD, LDAMV
Set 2	ASS, MFL	IEMG, LDAMV
Set 3	MAV, LDAMV	RMS, LDAMV
Set 4	MAD, LDAMV	DASDV, LDAMV
Set 5	MMAV, LDAMV	VO, LDAMV
Set 6	Vo, LDAMV	IQ, MFL
Set 7	IQ, MFL	LDAMV, MAV
Set 8	MAV, MFL	IQ, LDAMV
Set 9	IEMG, LDAMV	IEMG, MFL
Set 10	ASS, LDAMV	ASS, LDAMV

#### 3.3.2 Three-feature combinations

**Figure 5:**
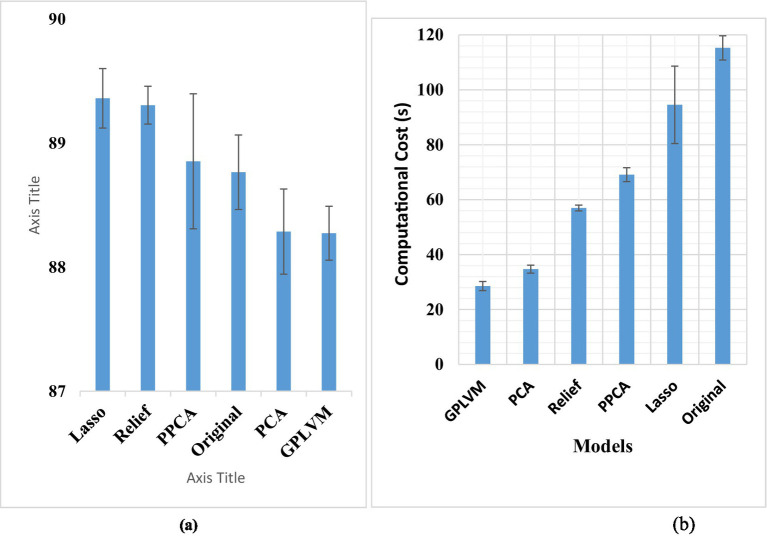
**(A)** Displays the classification accuracy, while **(B)** illustrates the computational time.

**Figure 6:**
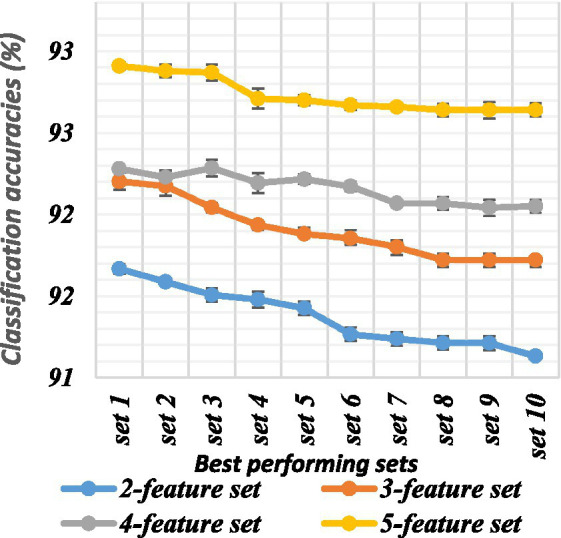
Classification accuracies of the top sets of all combinations for dataset 1.

There are a total of 560 combinations of the 16 features, each of which contains 3 features. All feature sets were evaluated using an ANN. The classification accuracy ranged from 85.58 to 92.40%. The best-performing feature sets were chosen based on the classification accuracies. The top 10 sets from both datasets are shown in Table 3. Their classification accuracies are plotted in Figures 5, 6.

**Table 3:** Optimal three feature sets.

Sets	Features	
	Dataset 1	Dataset 2
Set 1	VO, DAMV, LDAMV	MAV, DASDV, MFL
Set 2	MAV, MFL, LDAMV	STD, ASS, LDAMV
Set 3	IEMG, MFL, LDAMV	RMS, ASS, LDAMV
Set 4	IEMG, DASDV, LDAMV	MAD, DASDV, MFL
Set 5	MAD, DAMV, LDAMV	MMAV, DASDV, MFL
Set 6	MAV, DAMV, LDAMV	MMAV, DAMV, MFL
Set 7	MAV, STD, MFL	LD, DAMV, MFL
Set 8	LD, DAMV, LDAMV	RMS, MFL, LDAMV
Set 9	MMAV, IEMG, LDAMV	WL, ASS, LDAMV
Set 10	RMS, DASDV, LDAMV	IQ, MFL, LDAMV

**Figure 7:**
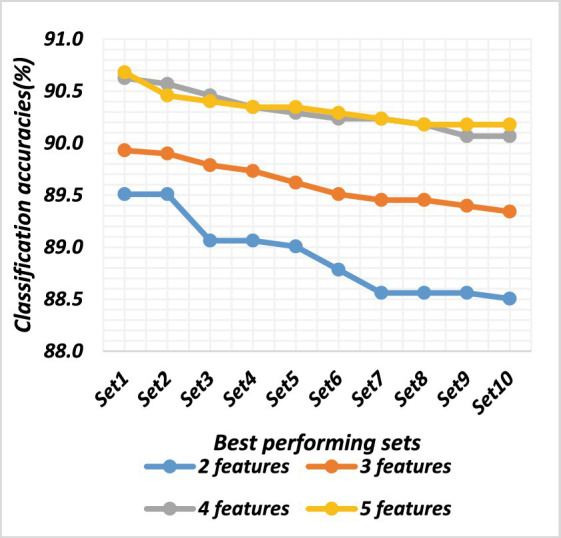
Classification accuracies of the top sets of all combinations for dataset 2.

#### 3.3.3 Four-feature combinations

There were a total of 1,820 combinations of the 16 features, each of which contained 4 features. All feature sets were evaluated using an ANN. The classification accuracy ranged from 85.37 to 92.48%. The best-performing feature set was chosen based on the classification accuracies. The top 10 sets of both datasets are shown in Table 4, and the classification accuracies are plotted in Figures 5, 6.

**Table 4:** Optimal 4 feature sets.

Sets	Features	
	Dataset 1	Dataset 2
Set 1	IEMG, Std, WL, LDAMV	MAD,IAV,MFL, LDAMV
Set 2	IAV, WL, DASDV, MFL	MAD,STD, DASDV, LDAMV
Set 3	IEMG, Vo, MFL, LDAMV	WL,DASDV, ASS, MFL
Set 4	RMS, WL, ASS, MFL	IAV,STD,ASS,LDAMV
Set 5	RMS, LDAMV,MAV, DASDV	IAV,STD, DASDV, MFL
Set 6	STD,WL, ASS, MFL	DAMV,WL, ASS, MFL
Set 7	MMAV, Vo, WL, DASDV	IAV,IEMG,WL, LDAMV
Set 8	MAD, DAMV, DASDV, ASS	IAV,RMS, DASDV,LDAMV
Set 9	IAV, Vo, MAV, DAMV	MAD,VO, STD, DAMV
Set 10	RMS, MAV, DAMV, LDAMV	MAV,DAMV,MFL,LDAMV

#### 3.3.4 Five-feature combinations

There were a total of 4,368 combinations of the 16 features, each of which contained 5 features. All feature sets were evaluated using an ANN. The classification accuracy ranged from 83.5 to 92.38%. The best-performing feature sets were chosen based on the classification accuracies. The top 10 sets are shown in Table 5. Their classification accuracies are plotted in Figures 6, 7.

**Table 5:** Optimal five feature sets.

Sets	Features	
	Dataset 1	Dataset 2
Set 1	MMAV, WL, DASDV,ASS, LDAMV	MMAV, RMS, MAV,ASS, LDAMV
Set 2	MFL, RMS, DAMV,LDAMV, MMAV	IQ, IEMG, MAV, MFL, LDAMV
Set 3	MMAV, MAD, IEMG, DASDV, LDAMV	IQ, MAD, RMS, MFL, LDAMV
Set 4	IQ, IAV,RMS, MFL, LDAMV	MMAV,LD,RMS, MFL, DAMV
Set 5	IQ,IEMG, RMS,DASDV, LDAMV	MMAV, MAV,WL, ASS, MFL
Set 6	IQ, Vo, MAV, DAMV, LDAMV	IQ, LD, DASDV, ASS, LDAMV
Set 7	IQ, WL, ASS, MFL, LDAMV	IQ, MAD, WL, ASS, MFL
Set 8	IQ, MAD, DASDV, MFL, LDAMV	IQ, LD, IEMG, DAMV, LDAMV
Set 9	IQ, Vo, DASDV, ASS, LDAMV	MMAV,IQ, RMS, ASS, LDAMV
Set 10	MMAV, MAD, WL, DASDV, LDAMV	MMAV, LD, STD, MFL, LDAMV

### 3.4 Comparison of feature sets

The classification accuracy of feature sets comprising 2, 3, 4, and 5 features was compared to determine the optimal number of features. ANOVA tests were conducted on both datasets to compare the accuracy of different feature sets, which were significantly different (*p* < 0.002). Both datasets exhibited statistically significant differences among all combinations (*p* < 0.05). The two-feature set significantly differed from the three-feature set (*p* < 0.05), and the three-feature set also showed significant differences compared to the four-feature set (*p* < 0.05), which, in turn, significantly differed from the five-feature set (*p* < 0.05). The average classification accuracy increased with an increase in the number of features in the set. In conclusion, the findings suggest that using a feature set comprising 5 features yields the highest classification accuracy in both datasets.

## 4 Discussion

This study highlights the emphasis of dimensionality reduction and feature selection. This study took a comprehensive approach by applying dimensionality reduction methods and exhaustive feature selection to the whole dataset. Unlike other works, which often use subject-specific techniques, our model is intended for use on a wide range of subjects and performs excellently regardless of one’s age or sex. This wider scope permits for a more adaptive, more generalized model that can be applied in many different situations and populations. Employing these advanced methodologies across the entire dataset created an all-inclusive robust model that overcame the restrictions set forth in subject-specific analyses.

We have developed an advanced and automated feature extraction method for improving our application. This was because we recognized that different data sets require unique set of features to attain optimum results, hence necessitating the use of a complex technique. It is designed to extract many features from data and subsequently rank them according to their usefulness in a given task. This enabled us to choose the most appropriate feature reducing time and thus improved over all accuracy and performance of our software intelligently through automation. Such inclusion of this automated approach for selecting features not only made it possible for us to address diverse datasets but also improved the robustness and reliability of our work; hence making our study a significant contribution toward development of feature extraction techniques (Figure 7).

Our investigation suggested that employing dimensionality reduction techniques such as PCA, GPLVM, lasso, relief, and PPCA can notably enhance both accuracy and reduce processing time. However, LDA may not be suitable for this type of analysis. Although these reduction techniques did not notably increase the classification accuracy, they significantly reduced the computational costs. They performed well, particularly improving classification accuracy when applied to larger datasets. Comparing each model individually, we found significantly lower computational times. The GPLVM had the shortest computational cost at 29 s, which was significantly less than that of all the other models (*p* < 0.05). PCA took approximately 35 s, which was significantly greater than that of GPLVM but notably less than that of the other models. Relief took approximately 57 s, which was significantly greater than that of GPLVM and PCA but less than that of the other models. PPCA took approximately 69 s, which was significantly greater than that of Relief, GPLVM, and PCA but less than that of the other models. Although the computational cost of Lasso was greater than that of all other models, it was lower than that of the original set.

**Figure 8:**
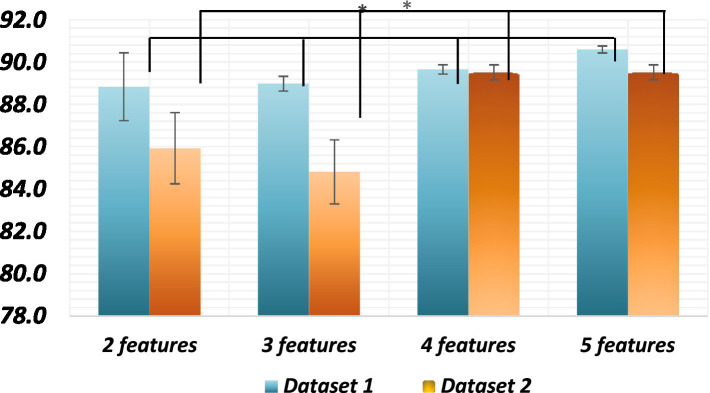
Average classification accuracies of all combinations* Significant differences between all sets of features of the datasets. The *p* value is less than 0.01.

All these models are viable for dimensionality reduction, with GPLVM emerging as the best model according to our study. However, for the highest accuracy, exhaustive feature selection remains the best model despite its high cost. The observed outcome might be attributed to the utilization of unsupervised dimensionality reduction techniques, which inherently aim to map high-dimensional data onto lower-dimensional spaces. Consequently, the selected features, while deemed optimal, may not possess substantial variance or discriminatory power in the reduced space. This could explain the observed limitation in capturing diverse patterns or distinguishing characteristics within the data (Figure 8).

**Figure 9:**
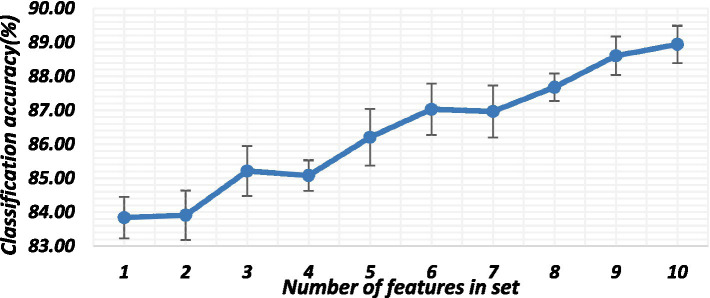
Number of features and their effect on classifier performance.

The number of features used and the types of considered features play a crucial role in the classification of hand gestures. This is why the features were initially ranked, and those performing poorly were subsequently eliminated from the set. Therefore, the number of features used depends on different factors, such as the type of activity, muscle, sampling frequency, and type of feature in a combination. Figure 9 illustrates the effect of the number of features on the classification accuracy. The classification accuracies generally show an upward trend from 2 to 10 features, followed by a slight decrease when there are more than 9 features in a set. This trend could be attributed to feature redundancy. In this work, we used exhaustive feature selection to obtain 10 high-performing sets of 2, 3, 4 and 5 combinations. Even though the protocols for both dataset acquisition and processing were the same, they still had different high-performing sets, as shown in Tables 2–4.

This study utilized data from 12 muscles to analyze five different movements. Among these, some muscles were identified as dominant for specific movements, while others were not. Even if a muscle is not dominant for a particular movement, it often plays a supporting or stabilizing role. The interplay between muscles is complex, as most movements require some degree of co-activation to ensure smooth and coordinated motion. To further reduce computational time, future studies could investigate whether classification accuracies remain consistent when the number of muscles analyzed is reduced or when only a subset of muscles is selected for the analysis.

## 5 Conclusion

This work provides evidence that by using dimensionality reduction techniques, higher-dimensional features extracted from multiple EMG channels can improve the accuracy of classification in EMG signal analysis. The use of such feature selection also significantly improved the classification accuracy, with the 5-feature set exhibiting the best performance. These findings have implications for developing more accurate and efficient algorithms for myoelectric control applications.

## Data Availability

The datasets presented in this study can be found in online repositories. The names of the repository/repositories and accession number(s) can be found below: https://doi.org/10.6084/m9.figshare.23898012.v1.
